# A community-based participatory research and community health workers-led health education strategy for schistosomiasis control among schoolchildren in Pemba Island, Zanzibar: a cluster-randomized trial

**DOI:** 10.1186/s40249-026-01504-y

**Published:** 2026-09-30

**Authors:** Xiaojing Li, Yi Wang, Mingzhen He, Saleh Juma Mohammed, Khamis Seif Khamis, Xinyao Wang, Xiangyu Zhou, Zhangyi Xu, Liang Shi, Mchanga Mohd Suleiman, Mgeni Abdalla Khamis, Yang Dai, Jie Sun, Kun Yang, You Li, Yuzheng Huang

**Affiliations:** 1 School of Public Health, Nanjing Medical University, Nanjing, China; 2 National Health Commission Key Laboratory of Parasitic Disease Control and Prevention, Jiangsu Provincial Key Laboratory on Parasite and Vector Control Technology, Jiangsu Institute of Parasitic Diseases, Wuxi, China; 3 Changzhou Center for Disease Control and Prevention, Changzhou, China; 4 Neglected Tropical Diseases Program, Ministry of Health Zanzibar (MoH), Pemba, Zanzibar, United Republic of Tanzania; 5 Department of Laboratory Medicine, Tongji Hospital, Tongji Medical College, Huazhong University of Science and Technology, Wuhan, China; 6 Animal and Plant Inspection and Quarantine Technology Center Shenzhen Customs, Shenzhen, China; 7 Department of Epidemiology, National Vaccine Innovation Platform, School of Public Health, Nanjing Medical University, Nanjing, China; 8 Key Laboratory of Public Health Safety and Emergency Prevention and Control Technology of Higher Education Institutions in Jiangsu Province, Nanjing Medical University, Nanjing, China; 9 Centre for Global Health, Usher Institute, University of Edinburgh, Edinburgh, UK; 10 Changzhou Medical Center, Changzhou Third People’s Hospital, Nanjing Medical University, Changzhou, China

**Keywords:** Schistosomiasis, Community health worker, Health education, Community-based participatory research, Tanzania

## Abstract

**Background:**

Schistosomiasis remains an intractable public health challenge in sub-Saharan Africa. While mass drug administration (MDA) has been the cornerstone of control, its limited impact on sustained behavioral change and persistent transmission in high-intensity transmission areas underscores the urgent need for complementary strategies. We aimed to evaluate the effectiveness of a community-based participatory research (CBPR) approach led by community health workers (CHWs), compared to conventional health education, in achieving sustainable schistosomiasis control among schoolchildren in Pemba, Zanzibar.

**Methods:**

A pilot, cluster-randomized controlled trial (cRCT) was conducted from February 2024 to February 2025. Since the screening revealed that the infection rates in all six local schools exceeded 3%, we randomly selected two of them and randomized them 1:1 to receive either a CBPR-CHW health education (1 intervention school) or conventional health education (1 control school). A total of 650 eligible school children aged 11–13 years were initially enrolled. After screening, 602 children completed the baseline survey and were assigned to either the CBPR-CHW intervention group (*n* = 278) or the control group (*n* = 324). After accounting for attrition, the final analytical sample at the 12-month follow-up (T3) consisted of 540 participants. The CBPR intervention was developed through a structured process of community needs assessment and co-design culminating in six culturally adapted educational modules delivered by trained CHWs. Primary outcomes were *Schistosoma haematobium* infection prevalence (urine egg positivity) and comprehensive knowledge, attitudes, and practices (KAP) scores, assessed at baseline (T1), 6 months (T2), and 12 months long-term follow-up (T3). Analyses utilized two-way repeated-measures ANOVA with Bonferroni correction for KAP score comparisons, supplemented by Kruskal–Wallis tests for non-normal data. Missing values (< 5%) were addressed via multiple imputation.

**Results:**

Loss to follow-up was low across all time points, ranging from 8.5 to 11.9%. At the 12-month follow-up (T3), the CBPR-CHW group demonstrated a significantly greater reduction in infection prevalence (from 19.18 to 0.82%) (*P* < 0.001) compared to the control group (from 11.86 to 1.02%). The CBPR-CHW group also showed sustained, significant improvements in KAP scores, significantly outperforming the control group at T3 (e.g., knowledge scores: 7.11 ± 1.35 vs. 6.56 ± 1.70; *P* < 0.05). In contrast, the control group showed transient or non-significant improvements. High student satisfaction was reported (total score: 5.28 ± 1.28/6), and household latrine usage increased by 56.3%.

**Conclusions:**

The CBPR-CHWs-led strategy was significantly more effective than conventional education in achieving sustainable reductions in schistosomiasis infection and improvements in KAP outcomes. This model represents a highly effective, scalable complement to MDA, directly addressing gaps in the WHO’s 2030 elimination roadmap by fostering community ownership and cultural relevance.

*Trial registration*: ClinicalTrials.gov NCT06312462. Registered on February 28, 2024. Available at: https://register.clinicaltrials.gov/

**Supplementary Information:**

The online version contains supplementary material available at https://doi.org/10.1186/s40249-026-01504-y.

## Background

Schistosomiasis is a parasitic infection caused by *Schistosoma* trematodes found in tropical and subtropical climates, particularly in sub-Saharan Africa [1, 2]. It constitutes a major public health burden, endemic in 79 countries with preventive chemotherapy required in 50 countries. An estimated 253.7 million people required preventive chemotherapy globally in 2024, of whom 93.9% reside in Africa [3]. In countries like Tanzania, schistosomiasis remains endemic, with persistent transmission hotspots illustrating the ongoing challenge. For instance, parts of Pemba Island have seen prevalence resurge rapidly following intervention campaigns [4].

In response to this global challenge, the World Health Organization (WHO) has set a goal to eliminate schistosomiasis as a public health threat by 2030. The recommended strategy emphasizes an integrated approach that combines preventive chemotherapy, primarily through mass drug administration (MDA) of praziquantel, with complementary measures including water, sanitation, and hygiene (WASH) improvements, environmental management, and health education [5, 6]. However, current control efforts in many settings, including Pemba Island, have predominantly focused on MDA, often at the expense of sustained snail control and robust behavioral interventions [7]. This over-reliance on chemotherapy alone faces limitations, as evidenced by rapid reinfection rates in some areas, underscoring the need for more comprehensive strategies. Preventive chemotherapy must be effectively integrated with information, education, and communication (IEC) strategies to reduce high-risk water exposure and reinforce sustainable behavioral change [1, 8–10].

Health education is a systematic, cost-effective approach that empowers communities with the knowledge, skills, and motivation to adopt protective behaviors [11]. It bridges a critical gap in MDA-centric strategies by fostering long-term behavioral change, such as reducing contact with infested water [1, 12]. Nonetheless, conventional health education programs often face challenges related to sustainability and consistency. They are frequently implemented on an ad hoc basis by personnel who are themselves key community figures (e.g., teachers, community doctors, or primary healthcare workers). However, these individuals may lack specific, standardized training and dedicated resources for schistosomiasis control, limiting the long-term impact of such programs [13]. Furthermore, this approach can fail to address the sociocultural embeddedness of water-contact behaviors (e.g., for domestic use, livelihood, or recreation), which is a key driver of transmission. The WHO guidelines on community engagement highlight that sustainable prevention requires moving beyond information dissemination to empower communities and adapt interventions to local contexts [14].

To address these gaps, a shift towards community-driven, participatory approaches is warranted. Community-based participatory research (CBPR) is a collaborative approach that equitably engages community members, organizational representatives, and researchers in all phases of the research process, with the aim of combining knowledge and action for social change and improved health outcomes [15, 16]. This methodology was selected for its proven effectiveness in ensuring cultural relevance and fostering community ownership, which are critical for sustaining behavioral change in public health interventions [17, 18]. Such approaches position community members not as passive recipients, but as active co-designers and implementers of interventions, thereby enhancing relevance, acceptance, and ownership. Community health workers (CHWs) serve as vital frontline actors in the control of infectious diseases. In Zanzibar, CHWs are local community members selected by their communities and trained by the Ministry of Health to deliver basic health services and education. It is important to note that the CHWs involved in this study were community-elected volunteers. Functionally, they are similar to Community Directed Distributors (CDDs) widely used across Africa for NTD control, rather than government-employed and salaried Community Health Extension Workers (CHEWs) or Community Health Officers (CHOs) [19–21]. Their success in reducing malaria burden in settings like Myanmar and Cambodia through trusted, community-embedded service delivery and education demonstrates the potential of implementing health interventions within community-based structures [22–24]. We therefore employed a CBPR framework, partnering with these locally embedded CHWs, to develop and implement a culturally compelling and sustainable health education model for schistosomiasis control.

Furthermore, the success of such community-embedded strategies has the direct potential to strengthen primary healthcare (PHC) systems and achieve WHO’s NTD 2030 goals. By empowering frontline health workers and fostering community ownership, this approach aligns with global calls for sustainable, integrated interventions that move beyond vertical disease control. This study aimed to develop and evaluate a community-driven health education intervention to address gaps in current schistosomiasis control. We hypothesized that a CBPR-CHWs-led model would yield more feasible, culturally appropriate, and sustained behavioral outcomes than conventional top-down education. To empirically test this, we codesigned the intervention with local communities in Tanzania, positioning CHWs as facilitators of participatory learning. We then evaluated the feasibility and sustained impact of this CBPR-CHWs-led intervention among schoolchildren, with the detailed CBPR methodology described in the Methods section. This study offers critical insights into a scalable, community-embedded strategy for achieving lasting schistosomiasis control.

## Methods

### Study design and sample size calculation

This pragmatic pilot cluster-randomized controlled trial (cRCT) was conducted from February 2024 to February 2025 to evaluate the impact of a community-based participatory health education intervention on *Schistosoma haematobium* infection and knowledge, attitude, and practice (KAP) among schoolchildren. The trial protocol was registered at ClinicalTrials.gov (NCT06312462). We used the following formula to determine the sample size [25, 26]:

$$N\, = \,\frac{{Z^{2} \, \times \,P\,\left( {1\, - \,P} \right)}}{{\delta^{2} }}\, \times \,deff$$

where *N* = sample size; *Z* = *Z* statistic for confidence level; *P* = expected proportion prevalence rate; δ = allowable error. To account for the cluster randomized controlled trial (cRCT) design, we incorporated a design effect (*deff*) to adjust for clustering at the school level. Based on the expected prevalence rate of 1.2% (*P* = 0.012) [27], an allowable error (δ) of 2% (i.e., the ‘δ’ was estimated at 0.02) [28], and a 95% confidence level (*Z* = 1.96), the investigators presented their results with 95% confidence intervals (CI) and *deff* = 2 (i.e., where ‘ρ’, the intra-cluster correlation coefficient, gauged the homogeneity characteristics within the cluster, and ‘M’ represented the cluster size; the ‘ρ’ was estimated at 0.02 and ‘M’ at 51 in our study. Thus, $deff = 1 + \rho (M-1) = 1 + 0.02 \times (51-1) =2)$ [27, 28]. The calculated sample size was 228. To ensure adequate power and account for potential attrition, non-response, and sampling error, the sample size was expanded, resulting in a total of 602 participants enrolled at baseline (278 intervention school and 324 control school participants).

### Study setting and study population

The trial was conducted in two primary schools located in high-burden districts on Pemba Island, Zanzibar, Tanzania, where there is a region characterized by hilly inland areas and coastal flatlands with abundant freshwater bodies that provide ideal habitats for intermediate host snails [27, 29]. Despite decades of school-based MDA with praziquantel, persistent transmission and recurrent infections remain prevalent [4], underscoring the need for sustainable complementary interventions. Rural schoolchildren are particularly at risk due to frequent contact with unsafe water sources (e.g., wells, springs, and streams) for domestic and recreational activities [30–32].

The study population comprised permanent resident schoolchildren aged 11–13 years (grades 4–6). This age group represents a critical window for habit formation, possessing the cognitive capacity for concrete operational thought necessary to understand disease transmission causality, while simultaneously exhibiting high infection risk due to water-contact behaviors. Inclusion criteria required participants to be aged 11–13 years, enrolled in grades 4–6, permanently residing in the school catchment area for ≥ 2 years, and providing written informed consent from a parent/guardian plus assent. Exclusion criteria included severe cognitive or communication impairments, temporary enrollment status, and incomplete baseline data..

### Randomization, allocation and masking

Schools were initially screened using historical surveillance data from the Zanzibar NTD program; six schools with a documented *S. haematobium* prevalence > 3.0% were identified. From these, two schools were randomly allocated in a 1:1 ratio to either the intervention or control arm following agreement from school authorities and community leaders. Randomization was performed at the school level (cluster) by an independent statistician, with allocation concealed from the research team responsible for enrollment and baseline assessments until finalized. Due to the nature of the behavioral intervention, masking of study personnel (including community health workers, researchers, and laboratory technicians) was not feasible; however, participating students were not explicitly informed of their specific group assignment. The intervention arm received CBPR-CHW-led health education, while the control arm received conventional health education delivered by Primary Health Care (PHC) staff.

### Study interventions

#### Intervention arm: community-based participatory research (CBPR) with CHWs

**Fig. 1:**
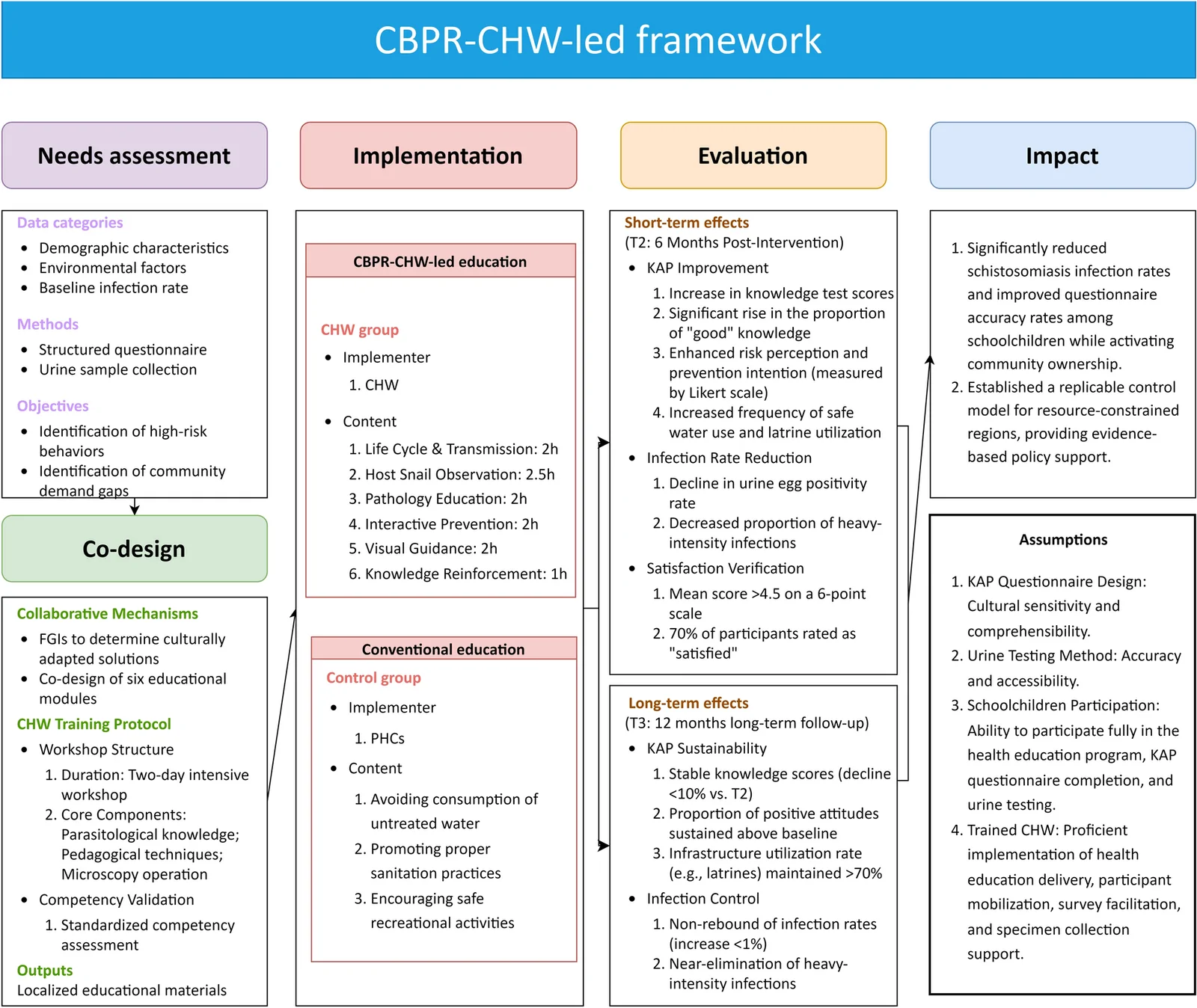
Schematic diagram of the CBPR-CHW-led health education intervention. The process begins with a needs assessment to gather community data, leading to the co-design phase where localized educational modules are collaboratively developed. The implementation phase contrasts the intervention (led by trained CHWs using participatory modules) with the control (standard health education by PHCs). The evaluation phase assesses short-term effects (at 6 months) and long-term effects (at 12 months) on knowledge, attitudes, practices, and infection rates. The final impact summarizes the expected outcomes: reduced infections and a replicable control model. The bottom section lists the underlying assumptions supporting the framework. CBPR: Community-based participatory research; CHW: Community health worker; PHC: Primary health center; FGI: Focus group interview; KAP: Knowledge, attitude, practice

This study employed a CBPR approach, defined as a collaborative partnership that actively engages local communities as equal partners in all research phases [33, 34]. This methodology was chosen to enhance cultural relevance, foster community ownership, and ensure the sustainable adoption of preventive behaviors. To implement this approach, we trained CHWs as trusted frontline facilitators. The intervention was developed and delivered through a structured CBPR cycle (Fig. 1):

1. Needs assessment & co-design: Prior to intervention delivery, three semi-structured focus group interviews were conducted with key stakeholders (students, parents, community elders, local experts; *n* = 32; see Additional file 1). These sessions identified context-specific exposure risks and preferences, which informed the content and delivery methods.
2. CHW training & role: In this study, CHWs were engaged throughout all phases of the project. During the pre-implementation stage, they assisted the research team in establishing connections with the community, were responsible for organizing focus group discussions, and facilitated communication between community members and researchers. All eight CHWs participated in a comprehensive 2-day workshop covering schistosomiasis knowledge, participatory teaching methods, and the use of educational aids. A key component was training in microscopy for teaching demonstrations, enabling them to use pre-screened local snail samples to show students the parasite life cycle, an activity intended solely for educational purposes, not for diagnosis. During the 6-month intervention period, CHWs served as facilitators for all interactive sessions (e.g., guiding games, conducting scenario simulations). In the follow-up assessment phase, they assisted with logistics, supported community mobilization, and administered questionnaires using a cross-over approach (i.e., CHWs from the intervention school collected data in the control school, and vice versa) to minimize assessment bias. The CHWs’ roles in this study, including health education, community mobilization, and supportive data collection, were extensions of their routine roles within Zanzibar’s primary healthcare system, while enhancing the workers disease control skills.
3. Implementation: The intervention was delivered monthly over a period of 6 months during regular school hours, scheduled in coordination with school authorities to minimize disruption to core academic instruction. Based on co-design outputs, six interactive, evidence-based educational modules were developed, with each session representing one module.

Module 1: Life cycle & transmission (2 h): schistosomiasis transmission pathways were explained via water source photos and exposure behavior analysis.

Module 2: Host snail observation (2.5 h): microscopic examination of local snails combined with a ‘Parasite Prevention’ comic creation competition.

Module 3: Pathology education (2 h): The infection process was demonstrated via anatomical models and guided symptom self-checking.

Module 4: Interactive prevention training (1.5 h): Implemented quiz games and high-risk behavior scenario simulations.

Module 5: Visual guidance reinforcement (2 h): Display posters on safe water use and hygiene practices.

Module 6: Knowledge reinforcement (1 h): Distributed health education booklets.

#### Control arm: conventional health education

The control arm received the standard, didactic health education typically delivered in school settings by Primary healthcare center (PHC) staff. The content in each session focused on avoiding consumption of untreated water, promoting proper sanitation practices, and encouraging safe recreational activities. This was delivered as a series of sessions to match the intervention group’s contact time: one session per month over 6 months, with each session being a single lecture lasting approximately 1–2 h. The sessions were delivered by PHC staff and did not contain any participatory or co-designed elements. This conventional health education was not delivered in the intervention school.

#### Standard care for both arms

All participants in both study arms continued to be eligible for school-based MDA with praziquantel as part of the Zanzibar Neglected Tropical Diseases Program. In adherence to ethical guidelines, all students who tested positive for *S. haematobium* at any survey point (T1, T2, or T3) were provided with praziquantel treatment promptly after their results were confirmed. However, no school-wide MDA round occurred during the entire study period (i.e., between T1 and T3). The last MDA round preceded the baseline survey (T1) by approximately 6 months, and the subsequent round was conducted after T3.

### Study outcome measures

The primary outcomes were: the prevalence of *S. haematobium* infection (urine egg positivity rate) at the 6-month (T2) and 12-month (T3) follow-ups, compared between the intervention and control arms; and the difference in changes in KAP scores from baseline (T1) to the 6-month (T2) and 12-month (T3) follow-ups between the intervention and control arms. Knowledge was scored from 0 to 10 points (1 point per correct answer), and attitudes were evaluated via a 5-point Likert scale with a maximum score of 55 points. Behavioral patterns were scored according to frequency (1–3 points per item), totaling a maximum of 30 points [converted to percentages and categorized as poor (< 60%), moderate (60–80%), or good (≥ 80%)] [35]. The secondary outcomes included infection intensity classified according to the WHO criteria (light: 1–49 eggs/10 ml urine; heavy: ≥ 50 eggs/10 ml urine) [36], and student satisfaction with the CHW-led intervention, measured on a 6-point scale (1 = dissatisfied, 2 = neutral, 3 = satisfied per item).

### Data collection

Trained CHWs administered face-to-face questionnaires in Kiswahili to assess students’ KAP. To minimize bias, CHWs who delivered the intervention did not administer questionnaires to participants from their own intervention group; they were cross-assigned to assess control groups where possible. The questionnaires underwent a rigorous translation and adaptation process, including forward-translation from English to Kiswahili, independent backtranslation to English to ensure conceptual equivalence, and review by a bilingual panel for cultural appropriateness, followed by pilot testing and validation (see Additional file 2) [1, 29, 37, 38].

For biological sample collections, students provided first-morning urine samples (30–50 mL) in sterile, pre-labeled wide-mouth containers under teacher supervision to ensure protocol adherence [39]. The samples were transported under controlled conditions to the China-Zanzibar Pathogen Biology Laboratory on Pemba Island. Certified laboratory technicians performed standardized filtration and microscopic analysis to detect *S. haematobium* eggs. All egg counts were initially recorded manually and then entered into a Microsoft Excel version 2016 (Microsoft Corporation, Redmond, WA, USA) database via dual-entry verification to maintain data integrity.

### Data management and statistical analysis

The collected data, including demographic characteristics, parasitological outcomes (urine microscopy results), and KAP survey responses, underwent rigorous quality control with double-entry verification in Microsoft Excel version 2016 prior to analysis in IBM SPSS Statistics version 26.0 (IBM Corp., Armonk, NY, USA). For missing values (< 5%), multiple imputation was performed using the MICE algorithm. Demographic and KAP-related variables were summarized via descriptive statistics, with continuous data presented as the Means ± SDs for normally distributed variables or medians [IQRs] for nonnormally distributed variables. To evaluate between‑group differences and changes over time in schistosomiasis KAP scores, data from the intervention group and the control group were compared across the three assessment periods (baseline, post-intervention, and follow-up) using two-way repeated-measures ANOVA with Bonferroni correction for multiple comparisons. For ordinal data that violated ANOVA assumptions of normality or homogeneity of variance (as confirmed through Q-Q plots and Levene’s testing), nonparametric Kruskal‒Wallis tests were implemented as appropriate.

### Patient and public involvement

Members of the public, including students, parents, community elders, and local experts, were involved in the design and implementation of this research through the CBPR framework, contributing to needs assessment and intervention co-design; however, they were not involved in the reporting or dissemination of this study.

### Protocol adherence and deviations

The study was conducted in substantial accordance with the pre-approved protocol. No substantial amendments to the protocol were required or made after ethical approval. However, the following minor deviations occurred during implementation:

Terminology Standardization: The study personnel referred to in the protocol as “community health volunteers (CHVs)” are subsequently described via the standardized term “community health workers (CHWs)” throughout this manuscript. This change in terminology represents a minor deviation in reporting for the sake of consistency with prevalent literature and does not reflect any change in the roles, identities, or composition of the implementing personnel.

Intervention Delivery: The duration and specific interactive activities within the six educational modules (e.g., the exact format of the ‘quiz games’ and ‘scenario simulations’) were refined during the codesign workshops with community stakeholders to increase cultural appropriateness and engagement. This iterative improvement process was a core principle of our CBPR approach but represents a deviation from a completely fixed intervention protocol.

Outcome assessment: All outcomes were assessed as prespecified. No changes were made to the primary or secondary outcomes after the trial commenced.

### Ethical considerations

The study protocol was approved by the Zanzibar Ethics Review Committee (ZAMREC/002/MAY/014) and was registered at ClinicalTrials.gov (NCT06312462; https://register.clinicaltrials.gov/). All participants retained the right to withdraw from the study at any point without consequence, with strict confidentiality maintained through anonymized data collection and secure storage protocols. In accordance with ethical principles of benefit-sharing, control schools received the full CBPR intervention package following completion of the trial's outcome assessments. We used the CONSORT reporting guidelines [40] to draft this manuscript and the CONSORT reporting checklist when editing, which are included in the Additional file 3.

## Results

### Participant characteristics and baseline data

**Fig. 2:**
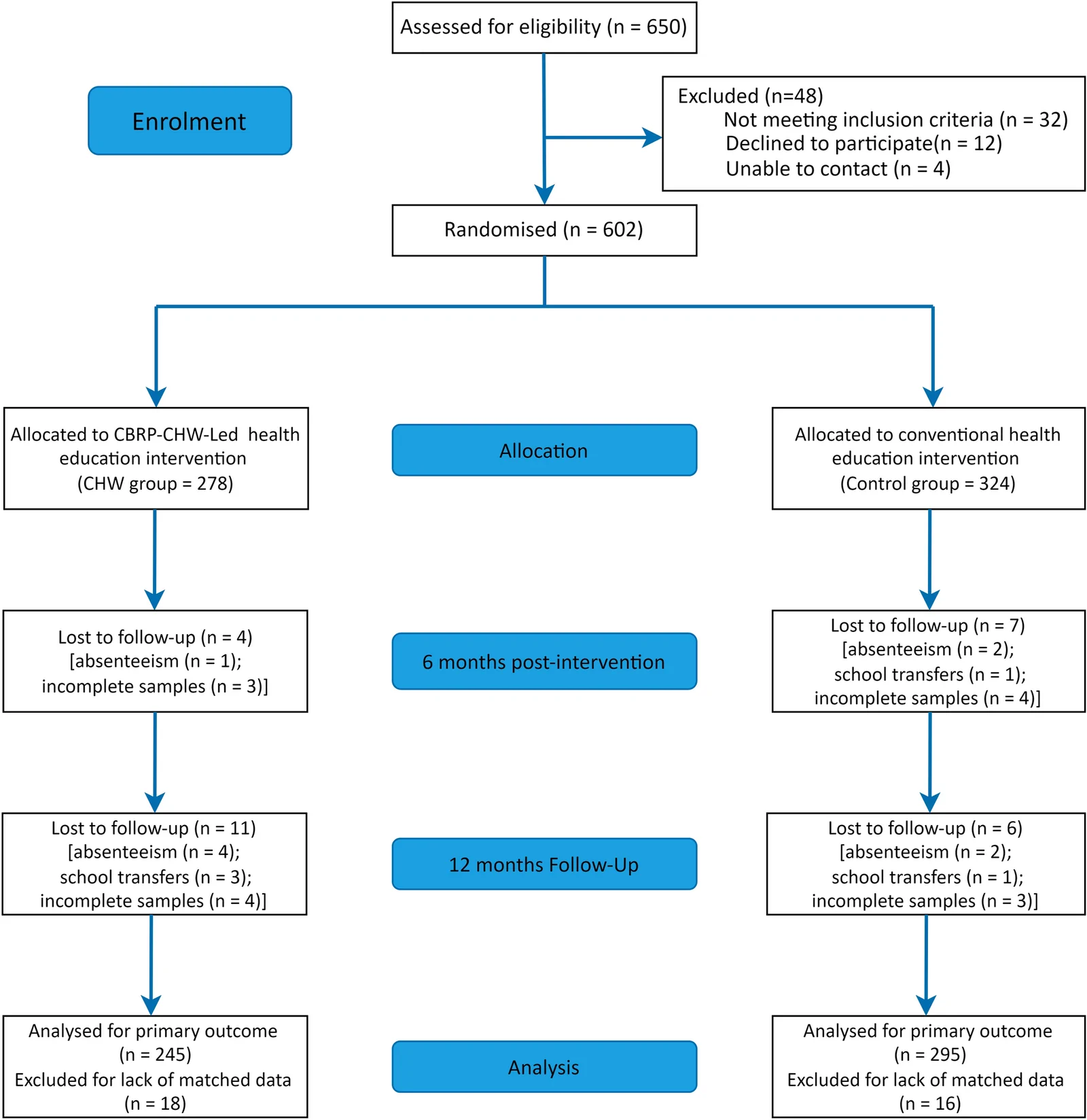
Participant flow diagram of the cluster-randomized trial. CHW: Community health worker

All 602 eligible students who were approached provided consent and were enrolled. Participant flow through the study is summarized in Fig. 2. Initial screening and enrollment were conducted in February 2024 (T1, baseline). A total of 602 eligible students from the two randomized schools were enrolled and assigned to study arms: the CBPR-CHW group (n = 278) and the control group (n = 324). After accounting for attrition due to school transfers, absenteeism on follow-up days, and the provision of incomplete biological samples, the final analytical sample at T3 consisted of 540 participants. The retention rates were 88.1% (n = 245) in the intervention group and 91.0% (n = 295) in the control group. Follow-up assessments were conducted at the participants’ school. Multiple attempts were made to reach absent students on subsequent days within a 2-week assessment window before they were classified as lost to follow-up.

Baseline demographic comparisons revealed well-balanced groups across all measured characteristics (all *P* > 0.05), including comparable age (mean 12.0 ± 1.4 years), gender distribution (40.0% male), household water/sanitation access, and parental occupation (see Supplemental Table 1).

### CBPR-CHWs intervention achieves near-elimination of Schistosoma haematobium infection

The *S. haematobium* infection rate demonstrated a statistically significant reduction over time within the CHW group (19.18% at T1; 2.45% at T2; 0.82% at T3; *Q* = 68, *P* < 0.001). The control group also exhibited a significant decline (11.86% at T1; 1.36% at T2; 1.02% at T3; *Q* = 47, *P* < 0.001).

**Fig. 3:**
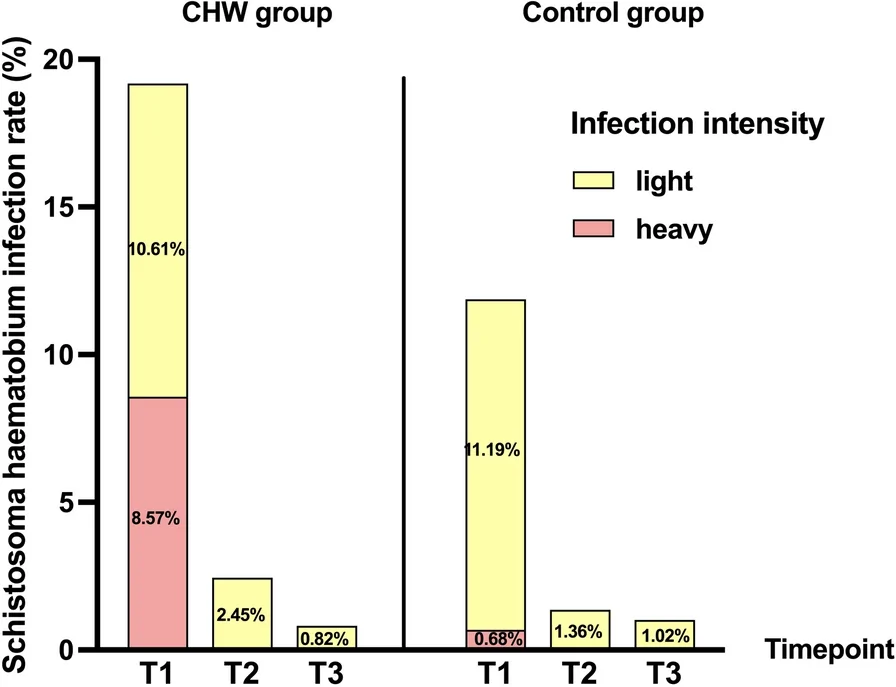
Prevalence of Schistosoma haematobium infection at T1/T2/T3 across two schools. The chart compares infection rates across three time points—baseline (T1), 6 months post-intervention (T2), and 12 months of follow-up (T3)—among the three study groups. CHW Group: Schools receiving the CBPR-CHW-led education intervention. Control Group: Schools receiving conventional health education. Stacked bars represent infection intensity and are segmented into light (1–49 eggs/10 ml of urine, yellow) and heavy (≥ 50 eggs/10 ml of urine, pink) infections. The CHW intervention group demonstrated a profound and sustained reduction in both overall prevalence and infection intensity, achieving near elimination (< 1%) by T3 with no rebound, outperforming both control groups. CBPR: Community-based participatory research; CHW: Community health worker

Analysis of infection intensity (Fig. 3) revealed a pronounced decline in the elimination of heavy infections from 8.57% at baseline (T1) to 0.82% at T3 exclusively in the CHW group. Both light and heavy infections decreased substantially for CHWs, with heavy infection dropping by nearly 90%, whereas the control group showed less marked light infection reduction.

### Reliability and validity of the KAP questionnaire and overall score trends

The questionnaire underwent reliability and validity testing, yielding a Cronbach’s alpha coefficient of 0.760, a KMO value of 0.785, and a significant Bartlett’s test of sphericity value of 2818.718 (*P* < 0.001), supporting validity for factor analysis. KAP outcomes were evaluated via two-factor repeated-measures ANOVA (Table 1) for continuous scores and the Kruskal–Wallis test for categorical levels (see Supplemental Table 2) across T1, T2, and T3.

**Table 1:** Repeated-measures ANOVA results for KAP scores across two schools

KAP	CHW group (Mean ± *SD*)	Control group (Mean ± *SD*)	*F*	*P*
Knowledge scores				
T1	6.11 ± 1.79	6.42 ± 1.56A	4.647	0.032
T2	7.13 ± 1.44a	6.62 ± 1.32A	18.222	< 0.001
T3	7.11 ± 1.35b	6.56 ± 1.70A	16.870	< 0.001
*F*	34.448	1.429		
*P*	< 0.001	0.240		
Attitude scores				
T1	38.02 ± 8.49	39.06 ± 8.96	1.922	0.166
T2	41.20 ± 8.66a	41.24 ± 8.15a	0.003	0.959
T3	43.58 ± 8.95bc	41.39 ± 8.58bA	8.357	0.004
*F*	27.751	7.311		
*P*	< 0.001	0.001		
Practice scores				
T1	19.05 ± 2.74	19.35 ± 2.87	1.490	0.223
T2	20.56 ± 2.84a	20.25 ± 2.64a	1.783	0.182
T3	20.70 ± 2.91b	20.14 ± 2.66bA	5.436	0.020
*F*	27.599	9.480		
*P*	< 0.001	< 0.001		

^*^Superscript letters denote statistically significant differences (*P* < 0.05) as follows: a and b vs. T1; c vs. T2; A vs. CHW group. All pairwise comparisons were adjusted via the Bonferroni method. CHW: Community health worker; KAP: Knowledge, attitude, practice; *SD*: Standard deviation

#### Sustained improvement in schistosomiasis knowledge among intervention participants

ANOVA revealed significant main effects for group (*F* = 8.455, *P* = 0.004) and time (*F* = 26.050, *P* < 0.001), alongside a significant group × time interaction (*F* = 12.884, *P* < 0.001). The CHW group presented a significant, sustained increase in knowledge scores from T1 (6.11 ± 1.79) to T3 (7.11 ± 1.35). The control group showed fewer stable patterns (decline at T3). Post hoc comparisons confirmed significantly higher knowledge scores in the CHW group than in the control group at T2 and T3 (all *P* < 0.05). The results of the categorical analyses revealed sustained improvement in the CHW group: the percentage of those with “good” knowledge increased significantly from 24.5% (T1) to 47.8% (T2) and 43.3% (T3) (*H* = 56.038, *P* < 0.001). Conversely, the control group showed no significant changes in the distribution of knowledge categories (*H* = 3.063, *P* = 0.216) (see Supplemental Fig. 1).

#### Positive shifts in health attitudes with long-term stability in the intervention group

While the intervention group’s main effect was non-significant (*F* = 0.567, *P* = 0.452), ANOVA confirmed a significant time main effect (*F* = 33.949, *P* < 0.001) and a significant group × time interaction (*F* = 5.726, *P* = 0.003). The CHW group achieved a significant increase in attitude scores from T1 (38.02 ± 8.49) to T3 (43.58 ± 8.95; *P* < 0.05), significantly exceeding the control group at T3 (both *P* < 0.05 post hoc). The control showed initial modest gains at T2, followed by stabilization at T3. Categorically, the CHW group displayed progressive improvement (*H* = 39.446, *P* < 0.001), with the proportion with “good” attitudes increasing from 26.7% (T1) to 51.4% (T3). Control group (*H* = 13.301, *P* = 0.001) also showed overall improvement by T3, albeit to a lesser extent than the CHW group did, which was particularly evident in the final attainment levels (see Supplemental Fig. 2).

#### Intervention group maintains improved preventive practices while control regress

While the group main effect was non-significant (*F* = 1.694, *P* = 0.194), a significant main effect for time (*F* = 36.966, *P* < 0.001), coupled with a significant group × time interaction (*F* = 3.648, *P* = 0.026), were found. The CHW group presented a significant increase in behavioral scores from T1 (19.05 ± 2.74) to T3 (20.70 ± 2.91; *P* < 0.05) and significantly outperformed the control group at T3. The behavior level analysis mirrored this finding: the CHW group achieved a significant reduction in “poor” behavior from 40.4% (T1) to 22.9% (T3) (*H* = 43.344, *P* < 0.001). The control group showed only transient improvement at T2 (poor behavior: 12.6% vs T1: 29.8%), relapsing toward baseline levels by T3 (17.0%; *H* = 27.22, *P* < 0.001) (see Supplemental Fig. 3).

### Marked increases in infrastructure-dependent protective behaviors following intervention

**Fig. 4:**
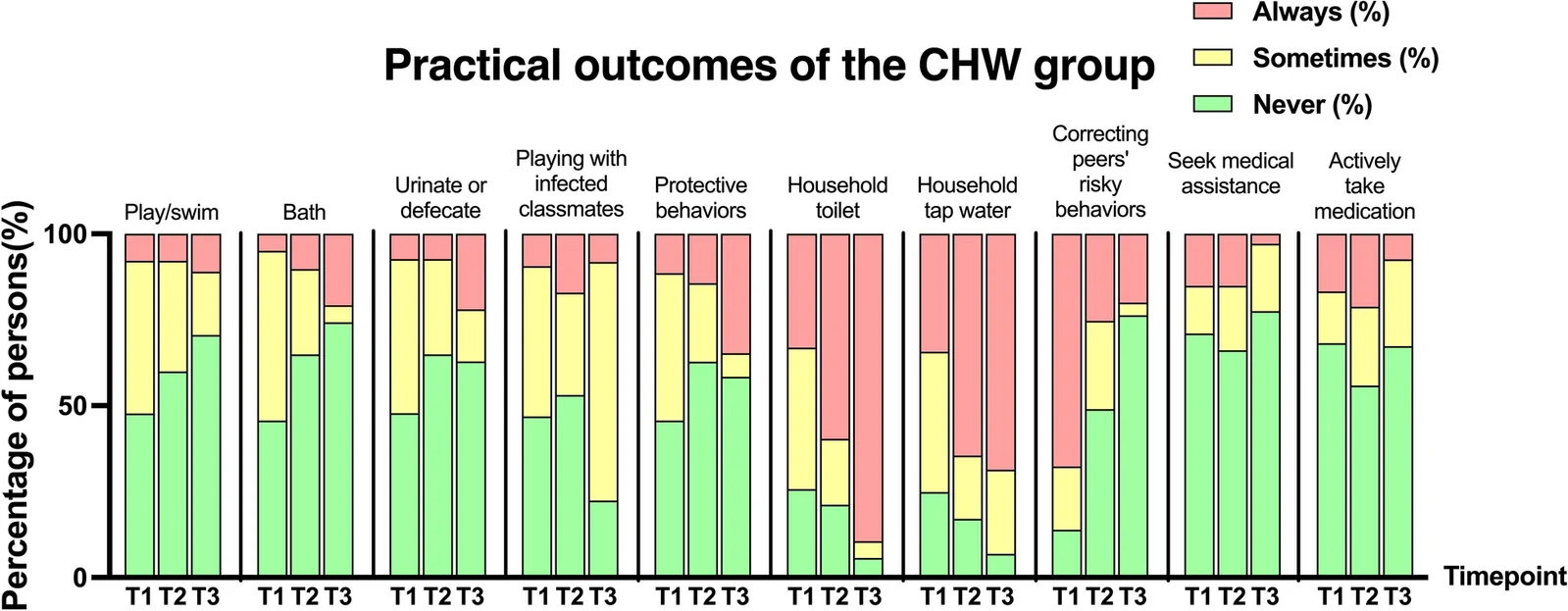
The CBPR-CHW intervention drove significant improvements in key preventive practices. This horizontal stacked bar chart details the self-reported practice frequencies for 11 specific behaviors across the three survey time points (T1 = Baseline, T2 = 6 months, T3 = 12 months) within the CHW intervention group. Response Metrics: For each behavior, the stacked bars show the percentage of respondents reporting the following: Always (Red): Consistent, ideal adherence to the preventive practice. Sometimes (yellow): Intermittent or occasional adherence. Never (Green): Consistent nonadherence or engagement in risky behavior. CBPR: Community-based participatory research; CHW: Community health worker

Data from the CHW group across three surveys (Fig. 4) revealed improvements in infrastructure-dependent health behaviors: the “always” rate for “household toilet use” increased from 33.1 to 89.4%, and “household tap water use” rose from 34.3 to 68.6%. Protective behaviors against exposure to natural water bodies (“Always”) increased from 11.4% at T1 to 34.7% at T3. However, risk behaviors related to natural water bodies showed a contradictory trend: while the proportion reporting “Never participating in water activities” increased, the “Always” participation rate also rose simultaneously. Among medical-related behaviors, initial improvements in healthcare-seeking and proactive medication-taking behaviors observed earlier did not persist until T3. In the social behavior dimension, the “always” rate for “playing with infected classmates” first increased but then decreased, whereas the “always” rate for “correcting peers’ risky behaviors” plummeted by 47.7 percentage points.

### High participant satisfaction with the CBPR-CHWs-led education program

The satisfaction assessment of the CBPR-CHWs health education interventions among the students revealed high acceptance and approval. The total satisfaction score (on a 6-point scale) was 5.28 ± 1.28, indicating strong overall satisfaction. Over 76% of the participants reported “satisfied” ratings for each individual evaluation item, reflecting consistent endorsement across all dimensions of the intervention.

## Discussion

This study demonstrated that the CBPR-CHWs-led health education intervention successfully achieved its primary aims, resulting in a profound and sustained reduction in *Schistosoma haematobium* infection rates and significant, durable improvements in KAP among schoolchildren. The longitudinal findings confirm the superior effectiveness and sustainability of this community-driven approach compared to conventional health education.

First, the CHWs intervention group’s knowledge scores rose significantly (from 6.11 to 7.13) and remained stable, whereas the control group’s scores fluctuated. This demonstrated that CHWs’ sustained engagement prevented a decline in knowledge that followed initial gains in the control group. Second, the CHW intervention group’s attitude scores improved (from 38.02 to 43.58), indicating that the intervention increased awareness of and willingness to adopt healthy behaviors. This study can help correct misconceptions and encourage prevention. In contrast, the control group’s short-term gains suggest that standard health education alone is not enough to change long-held beliefs, as seen in programs where knowledge gaps remain despite repeated efforts. Third, the CHW group maintained long-term improvements in healthy practices (from 19.05 to 20.70), whereas the control group declined slightly (from 20.25 to 20.14). This finding aligns with research indicating that lasting change requires both education and community involvement, not just passive lessons. Finally, the CHW group experienced a dramatic decrease in schistosomiasis infections (from 19.18 to 0.82%), which was linked to better knowledge and practices—especially increased toilet use (33.1–89.4%), reducing contaminated water exposure.

This study aligns with prior research demonstrating the effectiveness of participatory health education in schistosomiasis control [12, 41–43] but extends the evidence by showing how CBPR principles, when implemented through CHWs, can produce more durable outcomes than conventional approaches can. The sustained knowledge retention in our CHWs group contrasts with the decline in knowledge after initial gains seen in control groups, mirroring findings from Ndum et al. [42] in Tanzania, while surpassing their results through continuous CHWs engagement. Our improvements reflect the Health Belief Model’s (HBM) principles [44] and parallel Zaw et al.’s [45] success with CHWs in malaria programs, where trust-building proved critical for behavioral intent. The CHW group’s stable practice scores versus the control group regression support Torres-Vitolas et al.’s [1] conclusion that integrated, community-driven interventions outperform didactic methods. However, persistent challenges in protective behaviors and stigma reduction echo global findings [46, 47], underscoring the need for adaptive solutions beyond knowledge transfer. The infection rate decline (19.18–0.82%) surpasses typical MDA campaign outcomes [48], yet transient healthcare-seeking improvements, such as those of Feng et al. [49] and TGift [50], reveal that even robust CBPR models require systemic reinforcement through health system integration and policy alignment to sustain impact.

Regarding the generalizability and potential application of this CBPR-CHWs-led intervention, the model is promising for schistosomiasis control and potentially other NTDs in resource-limited settings globally, particularly in low- and middle-income countries with established CHWs infrastructure or strong community social capital. Regions that are culturally distinct from Tanzania or lack similar community health platforms, such as remote indigenous communities or densely populated urban informal settlements, warrant careful assessment for adaptation needs. Crucially, successful implementation elsewhere requires more than replicating the education program; key considerations include thorough contextual tailoring to address specific local beliefs and transmission dynamics, substantial investment in CHWs training and ongoing support mechanisms to ensure competency and motivation, and most critically, concurrent strengthening of the enabling environment. This involves ensuring reliable access to WASH, functional referral systems with available diagnostics and treatments at health facilities, and explicit policy integration [e.g., inclusion in Universal Health Coverage (UHC) schemes]. The intervention itself cannot compensate for systemic weaknesses in these areas, which emerged as significant constraints to achieving fully sustainable outcomes within this study.

This research has several notable strengths. Primarily, this study provides robust evidence for the effective implementation and impact of a rigorously adapted CBPR approach within a specific African context, addressing a significant gap in the literature on sustainable NTD control strategies. The longitudinal design, capturing changes across three timepoints, effectively demonstrated the sustained effectiveness of the intervention over time, with key outcomes largely maintained at the 12-month follow-up. Furthermore, the utilization of both KAP metrics and biological infection endpoints provided a comprehensive evaluation of the intervention’s effectiveness. Beyond schistosomiasis control, the CBPR-CHW model offers a scalable framework for strengthening community-based health systems. Its principles of co-design and local facilitation are suitable for integration into national NTD programs and school health curricula under the Ministries of Health and Education. This integration is crucial for leveraging the transition from time-limited projects into sustained public health practice. Empowering CHWs within this framework enhances program accountability, coverage, and community ownership, which are key to achieving sustainable NTD elimination.

However, several limitations must be acknowledged. The reliance on self-reported data for behavioral practices introduces the possibility of social desirability bias. The 1-year follow-up period, while demonstrating sustained changes in core outcomes such as knowledge, practices, and infection rates, remains insufficient to definitively confirm long-term sustainability, especially for changes in healthcare-seeking behavior, which could be transient. The study design focused primarily on schoolchildren, potentially overlooking critical household- and community-level transmission dynamics that influence the overall disease burden. Finally, unmeasured environmental and ecological heterogeneity across the study villages may have persisted as confounding factors, and the lack of in-depth qualitative data limits our understanding of the nuanced drivers of reported satisfaction and the broader socioecological context shaping behaviors and barriers. Future research should extend follow-up periods, incorporate mixed-method designs to explore contextual facilitators and barriers in depth, explicitly include household and community dynamics in transmission models, investigate adaptations for diverse populations and settings (e.g., preschool children, adults, and urban/peri-urban areas), and rigorously evaluate strategies for integrating CBPR interventions within strengthened health systems and supportive policy frameworks to achieve sustainable schistosomiasis control.

## Conclusion

This study provides compelling evidence that an innovative CBPR-CHWs-led approach is feasible and effectively produces durable improvements in schistosomiasis-related KAP and infection rates among schoolchildren in endemic settings. Our findings establish that CBPR-CHWs-led health education drives sustainable behavior change, particularly through the continuous engagement of culturally competent CHWs, which overcomes the limitations of conventional campaigns. However, the intervention's long-term success and broader public health impact are inherently linked to supportive health systems and cross-sectoral collaboration. This entails ensuring reliable drug access, fostering WASH partnerships, formalizing CHWs’ surveillance roles, empowering CHWs to codesign protective gear, and monitoring supply chains. To achieve lasting impact and sustainable control, programs must develop affordable, community-specific solutions, including stigma reduction. These efforts should be integrated into multisectoral NTD programs and national health policies through UHC frameworks, supported by dedicated funding, thereby contributing directly to primary healthcare strengthening and the 2030 NTD elimination goals.

## Supplementary Information

Additional file 1. Semi-structured focus group discussion guide. The semi-structured interview guide used for focus group discussions with community health workers during the intervention development phase. It covers topics related to local schistosomiasis perceptions, water-contact behaviors, and acceptability of health education strategies.

Additional file 2. Knowledge, Attitude, and Practice questionnaire. The structured questionnaire administered at baseline, 6-month, and 12-month follow-ups to assess participants' knowledge of schistosomiasis transmission, preventive attitudes, and self-reported high-risk water-contact behaviors.

Additional file 3. The CONSORT reporting checklist. For checking that reports of randomised trials can be understood and used by everyone.

Additional file 4.

Additional file 5.

## Data Availability

The data presented in this study are available upon request from the corresponding author. The data are not publicly available due to privacy concerns.

## Abbreviations

CBPR: Community-based participatory research
FGI: Focus group interview
KAP: Knowledge, attitude, practice
MDA: Mass drug administration
CHWs: Community health workers
CDDs: Community directed distributors
CHEWs: Community Health Extension Workers
CHOs: Community Health Officers
PHCs: Primary health centers
UHC: Universal Health Coverage
