# Supplementary material for: A community-based participatory research and community health workers-led health education strategy for schistosomiasis control among schoolchildren in Pemba Island, Zanzibar: a cluster-randomized trial
Source: Infect Dis Poverty. 2026 Sep 30;15:106. doi: 10.1186/s40249-026-01504-y (PMC13625477; doi:10.1186/s40249-026-01504-y)
Supplement: Supplementary file 1 [file 40249_2026_1504_MOESM1_ESM.docx]

**Semi-structured focus group interview outline**

**I. Introduction and informed consent**

Hello everyone, thank you for taking the time to be here. My name is Li Xiaojing. I am with the research team from Nanjing Medical University and the Jiangsu Institute of Parasitic Diseases. We are partnering with the Zanzibar Ministry of Health and the local Pemba schistosomiasis control program. We are here today to listen to you. We are designing a new health education program for students on the island, and since you live here and know the community best, your ideas will help us make sure this program actually works for you.

Before we start, please know that this conversation is completely voluntary. You do not have to answer any question you are not comfortable with, and if you want to stop at any point, that is perfectly fine. There is no penalty for doing so. Everything we talk about today is private and will only be used to improve our health program. We will not use your names or link any comments back to you personally. Does everyone agree to participate and have your thoughts included in this project? If so, we will get started.

**II. Local schistosomiasis awareness and context-specific exposure risk identification**

**【Student groups】**

1. Where do you usually go to play, bathe, or wash clothes? Think about ponds, rivers, or streams. Have you noticed any small animals in the water?
2. Are you familiar with schistosomiasis, or bilharzia? Do you know any classmates who have had this illness? What about symptoms like blood in urine or stomach pain?
3. How do you think people get schistosomiasis? Which activities do you think put you most at risk?
4. How much time do you typically spend near water sources during your commute to school and after school?

**【Parent/Community elder groups】**

1. What do you see with the children regarding their habits in fresh water? Have you noticed any illnesses related to playing in water?
2. In your view, what are the main drivers of schistosomiasis transmission in this community? Are there any traditional prevention methods practiced locally?
3. Who do you think is most vulnerable to schistosomiasis in this community, and why?

**【Local health experts/teachers groups】**

1. Based on your experience, what are the high-risk exposure scenarios for schoolchildren on Pemba Island? How do these differ from adult exposures?
2. What have been the primary challenges in schistosomiasis control in recent years? How would you prioritize interventions targeting schoolchildren?

**III. Barriers to existing health education and acceptability**

**【Student groups】**

1. Have you ever learned about schistosomiasis at school or from the clinic? How was that done? Was it through classes, pamphlets, or home visits?
2. Did you find those lessons interesting? Were they easy to understand and helpful?
3. What did you dislike about them? Were they boring, hard to follow, or did they not seem relevant to your daily life?

**【Parent/Community elder groups】**

1. Have you seen or heard messages about schistosomiasis prevention before? Where did you see them?
2. How useful were those messages? Was it difficult to get to where they were being shared?
3. Would you be willing to let your children take part in more prevention activities? Do you have any worries about that?

**【Local health experts/teachers groups】**

1. What are the practical challenges with the current school-based mass drug administration with praziquantel and the health education that goes with it?
2. Who do you think should be leading health education in the community, and why?

**IV. Co-design preferences for intervention content and delivery formats**

**【Student groups】**

1. If you were in charge of designing a health lesson on schistosomiasis, what would you want to include? Should it cover safe ways to play in water, how to manage the disease, or how to support friends who are sick?
2. Which ways of learning do you like best? For example, hearing stories from volunteers, playing games or competitions at school, watching short educational videos, doing group crafts, or something else?
3. You know the Community Health Workers, or CHWs, who visit homes or work at the local clinic. How would you feel if they came to your school or club to teach about bilharzia? Do you think you would listen to them more or less than you would a teacher?
4. If we asked you to practice prevention at home, would you be willing to tell your parents about it? Would you like them to be involved?

**【Parent/Community elder groups】**

1. What content would you like included in health education activities? Should adult needs also be addressed?
2. When is the best time for these activities? Would weekends, after school, or market days work best for you?
3. If community or religious leaders spoke about health, would that make people more likely to follow the advice? Would you be willing to help pass on the information?
4. Are there any topics considered inappropriate for public discussion? Are there cultural customs we should observe?

**【Local health experts/teachers groups】**

1. What key points should the intervention focus on? Identifying snails, taking medicine correctly, or safe water practices?
2. What are the benefits of having CHWs lead this education? What specific training should they get?
3. How can we fit this content into the existing school curriculum without putting too much pressure on the students and teachers?
4. How should we measure if this intervention is working? By testing knowledge, checking infection rates, or observing changes in behavior?

**V. Implementation feasibility and sustainability considerations**

1. What problems might come up when we try to run this project? Think about time, trust, or getting around the island.
2. To keep these health education activities going for the long term, what support is needed? This could be materials, incentives for volunteers, or partnerships with local institutions.
3. Is there anything else important that we have not asked about?

**VI. Closing and acknowledgments**

We would like to sincerely thank you all for your time and for sharing your honest thoughts today. Your insights are incredibly valuable and will directly shape how we design these health education activities. As a small token of our appreciation, we have some soap and notebooks to give to each of you. We will also post updates about the project at the local health dispensary if you wish to follow our progress. Thank you again.
