# Supplementary material for: A community-based participatory research and community health workers-led health education strategy for schistosomiasis control among schoolchildren in Pemba Island, Zanzibar: a cluster-randomized trial
Source: Infect Dis Poverty. 2026 Sep 30;15:106. doi: 10.1186/s40249-026-01504-y (PMC13625477; doi:10.1186/s40249-026-01504-y)
Supplement: Supplementary file 2 [file 40249_2026_1504_MOESM2_ESM.docx]

**Survey on the Knowledge, Attitudes, and Practices Regarding Schistosomiasis for Students in Pemba Island**

Dear students,

We are from the China-aid Project of Schistosomiasis Control in Zanzibar, which aims to elimination of schistosomiasis in Pemba. This survey on the knowledge, attitudes, and practices related to schistosomiasis among students is crucial aspect of our project, helping us to assess the current status of health education regarding schistosomiasis.

Thank you for taking the time to participate in this survey. Your responses play a vital role in advancing schistosomiasis control in your country. Remember, there are no right or wrong answers. Please feel free to enjoy the survey and don't hesitate to reach out if you encounter any issues. Your cooperation is greatly appreciated!

**A. Personal Information**

1.No:_____________

2.Gender: ①Male	②Female

3.Age: ______ years old

4.Number of people living in your household（including yourself ）: ______

5.The average amount of time you spend in school each day: ______hours

6.Father’s occupation: ______

①Farmer/Fisherman	②Worker	③Self-employed worker/Businessman

④Teacher	⑤Government staff ⑥Medical staff

⑦Unemployed	⑧Others_____

7.Mother’s occupation: ______

①Farmer/Fisherman	②Worker	③Self-employed worker/Businessman

④Teacher	⑤Government staff ⑥Medical staff

⑦Unemployed	⑧Others_____

8.Whether they have access to tap water near their residence? ①Yes	②No

9. Whether they have access to toilets near their residence？	①Yes	②No

10.Are there rivers, ponds, or any other water bodies around where you live?

①Yes	②No

11.How do you obtain information related to schistosomiasis? (Multiple choices allowed):

①China aid projects	②Hospital/NTD/PHC doctors	③Radio/Television

④School or teachers	⑤Family or friends	⑥No idea

⑦Other: ______

**B. Knowledge**

**Basic knowledge**

1.Schistosomiasis is a serious parasitic disease that affects the health of the body.

A. True	B. False

2.What types of schistosomiasis are included?

A. Intestinal schistosomiasis	B. Urinary schistosomiasis

C. Both	D. No idea

3.Which is the most common symptom of schistosomiasis haematobium?

A. Cough	B. Diarrhea

C. Haematuria	D. Weakness

4.Schistosomiasis is both curable and preventable.

A. True	B. False

**Exposure**

5.Activities such as swimming, fishing and walking barefoot in ponds can lead to schistosomiasis infection.

A. True	B. False

6.To contact with the urine from a patient infected with *Schistosoma haematobium* can lead to schistosomiasis infection.

A. True	B. False

**Transmission**

7.Which of the following ways could you infect with schistosomiasis?

A. To contact water containing schistosomiasis worms.

B. To eat raw or undercooked fish/meat.

C. To eat unwashed vegetables.

D. Close contact with individuals infected with schistosomiasis.

8.Which of the following organisms can transmit schistosomiasis?

A. Fly	B. Snail	C. Mosquito	D. Insect

**Treatment**

9.The preferred treatment drug for schistosomiasis is

A. Abendazole	B. Artemisinin	C. Mebendazole	D. Praziquantel

10.Schistosomiasis patients can be infected again after treatment.

A. True	B. False

**C. Attitudes**

**Basic attitude**

1.Schistosomiasis is a prevalent disease in your area.

A. Strongly disagree	B. Disagree	C. Neutral	D. Agree	E. Strongly agree

2.Schistosomiasis is a disease that seriously threatens human health.

A. Strongly disagree	B. Disagree	C. Neutral	D. Agree	E. Strongly agree

3.Anyone, including yourself, can contract schistosomiasis.

A. Strongly disagree	B. Disagree	C. Neutral	D. Agree	E. Strongly agree

4.If you notice blood in your urine or feel unwell, you should go to the hospital immediately.

A. Strongly disagree	B. Disagree	C. Neutral	D. Agree	E. Strongly agree

**Exposure**

5.You should use protective measures such as wearing rubber shoes if you play in any type of water.

A. Strongly disagree	B. Disagree	C. Neutral	D. Agree	E. Strongly agree

6.You can't get schistosomiasis by drinking raw water.

A. Strongly disagree	B. Disagree	C. Neutral	D. Agree	E. Strongly agree

**Transmission**

7.Snail control is important for blocking schistosomiasis transmission and preventing infection.

A. Strongly disagree	B. Disagree	C. Neutral	D. Agree	E. Strongly agree

8.Avoiding public defecation and promoting the construction of more toilets can contribute significantly to prevent the spread of schistosomiasis.

A. Strongly disagree	B. Disagree	C. Neutral	D. Agree	E. Strongly agree

**Treatment**

9.If you are infected with schistosomiasis, you are willing to receive medication such as praziquantel.

A. Strongly disagree	B. Disagree	C. Neutral	D. Agree	E. Strongly agree

10.If you are infected with schistosomiasis, your family can afford treatment.

A. Strongly disagree	B. Disagree	C. Neutral	D. Agree	E. Strongly agree

11.You are willing to receive regular health education or training on schistosomiasis.

A. Strongly disagree	B. Disagree	C. Neutral	D. Agree	E. Strongly agree

**D. Practices (For the last six months)**

**Exposure**

1. Did you play/swim in a pond/river/lake?

A. Always	B. Sometimes	C. Never

2. Did you bath in a pond/river/lake?

A. Always	B. Sometimes	C. Never

3. Did you urinate or defecate in a pond/river/lake?

A. Always	B. Sometimes	C. Never

4. Did you play with classmates who have schistosomiasis?

A. Always	B. Sometimes	C. Never

**Transmission**

5. Did you take some protective measures such as wearing gloves, rubber shoes when playing or swimming in a pond/river/lake?

A. Always	B. Sometimes	C. Never

6. Did you use the toilet to defecate and urinate at home?

A. Always	B. Sometimes	C. Never

7. Did you or your family use tap water for bathing, washing clothes, or cooking?

A. Always	B. Sometimes	C. Never

8. Did you offer support and correct 'bad behavior' to classmates who infected with schistosomiasis?

A. Always	B. Sometimes	C. Never

**Treatment**

9. Did you seek medical assistance when you notice blood in your urine?

A. Always	B. Sometimes	C. Never

10.Did you actively take medication when infected with schistosomiasis?

A. Always	B. Sometimes	C. Never
