# Supplementary material for: A community-based participatory research and community health workers-led health education strategy for schistosomiasis control among schoolchildren in Pemba Island, Zanzibar: a cluster-randomized trial
Source: Infect Dis Poverty. 2026 Sep 30;15:106. doi: 10.1186/s40249-026-01504-y (PMC13625477; doi:10.1186/s40249-026-01504-y)
Supplement: Supplementary file 4 [file 40249_2026_1504_MOESM4_ESM.docx]

**Supplemental Table 1.** Baseline demographic characteristics of the study participants, compared between groups

| **Characteristics** | **CHW group** | **Control group** | ***t/χ^2^*值** | ***P*值** |
| --- | --- | --- | --- | --- |
| **Age** | 11.75±1.441 | 11.98±1.370 | -1.920 | 0.055 |
| **Family member** | 8.42±2.515 | 8.23±2.653 | 0.814 | 0.416 |
| **Schooltime** | 6.78±0.634 | 6.85±0.513 | -1.416 | 0.158 |
| **Gender** |  |  |  |  |
| Male | 100(40.8%) | 113(38.3%) | 0.353 | 0.552 |
| Female | 145(59.2%) | 182(61.7%) |  |  |
| **Tap water** |  |  |  |  |
| Yes | 150(61.2%) | 204(69.2%) | 3.726 | 0.054 |
| No | 95(38.8%) | 91(30.8%) |  |  |
| **Toilet** |  |  |  |  |
| Yes | 175(71.4%) | 232(78.6%) | 3.754 | 0.053 |
| No | 70(28.6%) | 63(21.4%) |  |  |
| **Waterbodies** |  |  |  |  |
| Yes | 125(51.0%) | 174(59.0%) | 3.434 | 0.064 |
| No | 120(49.0%) | 121(41.0%) |  |  |
| **Father's occupation** |  |  |  |  |
| Farmer/Fisherman | 178(72.7%) | 216(73.2%) | 11.331 | 0.125 |
| Worker | 16(6.5%) | 35(11.9%) |  |  |
| Self-employed worker/Businessman | 28(11.4%) | 22(7.5%) |  |  |
| Teacher | 11(4.5%) | 9(3.1%) |  |  |
| Government staff | 8(3.3%) | 8(2.7%) |  |  |
| Medical staff | 2(0.8%) | 0(0.0%) |  |  |
| Unemployed | 2(0.8%) | 3(1.0%) |  |  |
| Others | 0(0.0%) | 2(0.7%) |  |  |
| **Mother's occupation** |  |  |  |  |
| Farmer/Fisherman | 146(59.6%) | 202(68.5%) | 11.672 | 0.112 |
| Worker | 18(7.3%) | 25(8.5%) |  |  |
| Self-employed worker/Businessman | 45(18.4%) | 28(9.5%) |  |  |
| Teacher | 15(6.1%) | 18(6.1%) |  |  |
| Government staff | 2(0.8%) | 3(1.0%) |  |  |
| Medical staff | 2(0.8%) | 2(0.7%) |  |  |
| Unemployed | 12(4.9%) | 15(5.1%) |  |  |
| Others | 5(2.0%) | 2(0.7%) |  |  |

**Supplemental Table 2.** Comparison of KAP levels across groups at different time points

| **Group** | **KAP** | **Time** | **Poor(n%)** | **Moderate(n%)** | **Good(n%)** | ***H*** | ***P*** |
| --- | --- | --- | --- | --- | --- | --- | --- |
| **CHW group** | Knowledge | T1 | 103(42.0%) | 82(33.5%) | 60(24.5%) | 56.038 | <0.001 |
|  |  | T2 | 30(12.2%) | 98(40.0%) | 117(47.8%) |  |  |
|  |  | T3 | 57(23.3%) | 82(33.5%) | 106(43.3%) |  |  |
|  | Attitude | T1 | 44(18.0%) | 135(55.3%) | 65(26.6%) | 39.446 | <0.001 |
|  |  | T2 | 35(14.3%) | 97(39.6%) | 113(46.1%) |  |  |
|  |  | T3 | 12(4.9%) | 107(43.7%) | 126(51.4%) |  |  |
|  | Practice | T1 | 99(40.4%) | 131(53.5%) | 15(6.1%) | 43.344 | <0.001 |
|  |  | T2 | 38(15.5%) | 173(70.6%) | 34(13.9%) |  |  |
|  |  | T3 | 56(22.9%) | 147(60.0%) | 42(17.1%) |  |  |
| **Control group** | Knowledge | T1 | 84(28.5%) | 129(43.7%) | 82(27.8%) | 3.063 | 0.216 |
|  |  | T2 | 55(18.6%) | 155(52.5%) | 85(28.8%) |  |  |
|  |  | T3 | 80(27.1%) | 118(40.0%) | 97(32.9%) |  |  |
|  | Attitude | T1 | 55(18.6%) | 146(49.5%) | 94(31.9%) | 13.301 | 0.001 |
|  |  | T2 | 45(15.3%) | 110(37.3%) | 140(47.5%) |  |  |
|  |  | T3 | 47(15.9%) | 116(39.3%) | 132(44.7%) |  |  |
|  | Practice | T1 | 88(29.8%) | 183(62.0%) | 24(8.1%) | 27.220 | <0.001 |
|  |  | T2 | 37(12.5%) | 219(74.2%) | 39(13.2%) |  |  |
|  |  | T3 | 50(16.9%) | 211(71.5%) | 34(11.5%) |  |  |
