# Supplementary material for: A community-based participatory research and community health workers-led health education strategy for schistosomiasis control among schoolchildren in Pemba Island, Zanzibar: a cluster-randomized trial
Source: Infect Dis Poverty. 2026 Sep 30;15:106. doi: 10.1186/s40249-026-01504-y (PMC13625477; doi:10.1186/s40249-026-01504-y)
Supplement: Supplementary file 5 [file 40249_2026_1504_MOESM5_ESM.docx]

**Supplemental figure 1. Sustained improvement in schistosomiasis knowledge levels following the CBPR-CHW intervention.** Stacked bar charts depict the distribution of knowledge levels (categorized as poor, medium, or good) across the two study groups at baseline (T1), 6 months post-intervention (T2), and 12 months of follow-up (T3). The CHW intervention group demonstrated a substantial and sustained increase in the proportion of students who achieved 'good' knowledge (from 24.5% at T1 to 43.3% at T3), coupled with a concomitant decrease in 'poor' knowledge. In contrast, control group showed only marginal or transient improvements.

**Supplemental figure 2. Significant and sustained improvements in health-seeking attitudes following the CBPR-CHW intervention.** Stacked bar charts illustrate the distribution of attitudes toward schistosomiasis prevention (categorized as poor, medium, or good) across the three study groups at baseline (T1), immediately post-intervention (T2), and at the 12-month follow-up (T3). The CHW intervention group demonstrated a dramatic and progressive shift toward positive health-seeking attitudes. The proportion of students with a 'good' attitude more than doubled, from 26.7% at T1 to 51.4% at T3, whereas the proportion with a 'poor' attitude category was reduced by nearly three-quarters (from 18.0% to 4.9%). In stark contrast, control group exhibited transient improvements at T2 that failed to be sustained at T3.

**Supplemental figure 3. CBPR-CHW intervention induces a strong immediate behavioral response.** Stacked bar charts illustrate the distribution of attitudes toward schistosomiasis prevention (categorized as poor, medium, or good) across the three study groups at baseline (T1), immediately post-intervention (T2), and at the 12-month follow-up (T3). The CHW intervention group demonstrated a powerful, intervention-driven response. The prevalence of 'good' practices increased from 6.1% (T1) to 17.1% (T3). Control group exhibited minimal and nonsignificant fluctuations in 'Good' practices, which remained consistently low (11.5%).
